# Supplementary material for: Global hypo-methylation in a proportion of glioblastoma enriched for an astrocytic signature is associated with increased invasion and altered immune landscape
Source: eLife. 2022 Nov 22;11:e77335. doi: 10.7554/eLife.77335 (PMC9681209; doi:10.7554/eLife.77335)
Supplement: Figure 2—source data 1. [file elife-77335-fig2-data1.zip › Figure_2_source_data_1/Figure_2A/homerResults/motif19.similar.html]

motif19

## Information for motif19

A
C
T
G
A
T
C
G
A
C
G
T
A
C
T
G
A
C
G
T
A
T
G
C
G
A
T
C
A
G
T
C
A
C
T
G
C
T
A
G
  
Reverse Opposite:  

A
G
T
C
T
G
A
C
C
T
A
G
C
T
A
G
A
T
C
G
C
G
T
A
A
G
T
C
C
G
T
A
A
T
G
C
T
G
A
C
  

|  |  |
| --- | --- |
| p-value: | 1e-20 |
| log p-value: | -4.645e+01 |
| Information Content per bp: | 1.850 |
| Number of Target Sequences with motif | 26.0 |
| Percentage of Target Sequences with motif | 1.12% |
| Number of Background Sequences with motif | 3.9 |
| Percentage of Background Sequences with motif | 0.11% |
| Average Position of motif in Targets | 94.4 +/- 51.2bp |
| Average Position of motif in Background | 93.4 +/- 44.6bp |
| Strand Bias (log2 ratio + to - strand density) | 0.6 |
| Multiplicity (# of sites on avg that occur together) | 1.35 |
| Motif File: | file (matrix) reverse opposite |

### Similar de novo motifs found

|  |  |  |  |  |  |  |  |
| --- | --- | --- | --- | --- | --- | --- | --- |
| Rank | Match Score | Redundant Motif | P-value | log P-value | % of Targets | % of Background | Motif file |
| 1 | 0.634 | T A G C C G T A T A C G C T A G C A T G T C A G T G A C G T C A A T C G A T G C G C T A A C G T | 1e-19 | -44.368174 | 1.21% | 0.11% | motif file (matrix) |
| 2 | 0.861 | A C G T A C T G A C G T A G T C A G T C A G T C A C T G C T A G | 1e-8 | -19.690267 | 1.46% | 0.44% | motif file (matrix) |
